# Supplementary material for: A large-scale genomically predicted protein mass database enables rapid and broad-spectrum identification of bacterial and archaeal isolates by mass spectrometry
Source: Genome Biol. 2023 Dec 5;24:257. doi: 10.1186/s13059-023-03096-4 (PMC10696839; doi:10.1186/s13059-023-03096-4)
Supplement: Supplementary file 1 — Additional file 1: Supplementary Material 1. The set of Pfam models used for extracting coding proteins likely relevant to ribosomal proteins. Figure S1. Relationship between average nucleotide identity (ANI) and genome-wide peak matching (PM) score. Figure S2. Relationship between taxonomic distance and genome-wide peak matching (PM) score. Figure S3. Relationship between taxonomic distance and genome-wide peak matching (PM) score for three genome assemblies with among the highest number of genes, or predicted mass peaks, in the GPMsDB (Kosakonia sp002886105: 8,428 predicted mass peaks with m/z values of 2,000–20,000; Streptomyces violaceus: 8,375; Actinoplanes liguriensi: 8,171). Figure S4. Effect of scoring scheme on identification accuracy as evaluated with simulated mass peak lists. Figure S5. Accuracy of identification using scoring scheme I for simulated peak lists with varying degrees of “noise”. Figure S6. Accuracy of identification using scoring scheme III for simulated peak lists containing varying numbers of ribosomal protein peaks. Figure S7. Schematic of the GPMsDB-tk. Figure S8. Identification results for MALDI-TOF MS peak lists of 71 bacterial reference strains. Figure S9. Identification results for MALDI-TOF MS peak lists of 10 archaeal strains. Figure S10. Identification results for MALDI-TOF MS peak lists of 13 strains of Actinomycetota. Figure S11. Identification results for MALDI-TOF MS peak lists for 74 different Acinetobacter strains. Figure S12. Same as for Figure S11, except that all genomes in the GPMsDB were searched by specifying command line option -a as “all”. Figure S13. Identification results for MALDI-TOF MS peak lists for 24 different strains of Cutibacterium acnes. [file 13059_2023_3096_MOESM1_ESM.pdf]

Supplementary Material 1. The set of Pfam models used for extracting coding proteins likely relevant to ribosomal proteins.

PF01199, PF01247, PF01907, PF00338, PF01780, PF00276, PF00935, PF01667, PF01020, PF01248, PF00831, PF00366, PF00238, PF00253, PF00410, PF01246, PF01200, PF01092, PF01282, PF01599, PF00411, PF17135, PF00380, PF01201, PF00833, PF01775, PF01198, PF00832, PF01157, PF16906, PF00428, PF00076, PF01655, PF01781, PF01294, PF00298, PF04758, PF01283, PF03297, PF00828, PF00203, PF00573, PF01090, PF00572, PF03946, PF00687, PF00327, PF00467, PF00297, PF00312, PF00237, PF00673, PF17144, PF07650, PF03719, PF00827, PF00281, PF00181, PF03947, PF08069, PF00164, PF01015, PF00416, PF00347, PF01280, PF00252, PF08071, PF01479, PF00177, PF00189, PF00466, PF17777, PF14204, PF00318, PF00333, PF03948, PF16205, PF12172, PF13240, PF01165, PF00163, PF00641, PF00900, PF01250, PF01084, PF16320, PF00453, PF17136, PF00861, PF00240, PF00444, PF01783, PF01245, PF00830, PF06698, PF01929, PF00468, PF01197, PF00424, PF00471, PF13302, PF01632, PF14520, PF01158, PF01196, PF00583, PF00829, PF01016, PF01649, PF00886, PF00575, PF01281, PF01386, PF04839, PF14693, PF17070, PF00542, PF01779, PF08007, PF04266, PF03059, PF00733, PF16551, PF00213, PF04327, PF09809, PF13113, PF11154, PF03143, PF11573, PF01776, PF07498, PF13541, PF09128, PF02684, PF12974, PF09657, PF00497, PF13420, PF17067, PF13673, PF00404, PF12949, PF13275, PF05794, PF01594, PF14374, PF01251

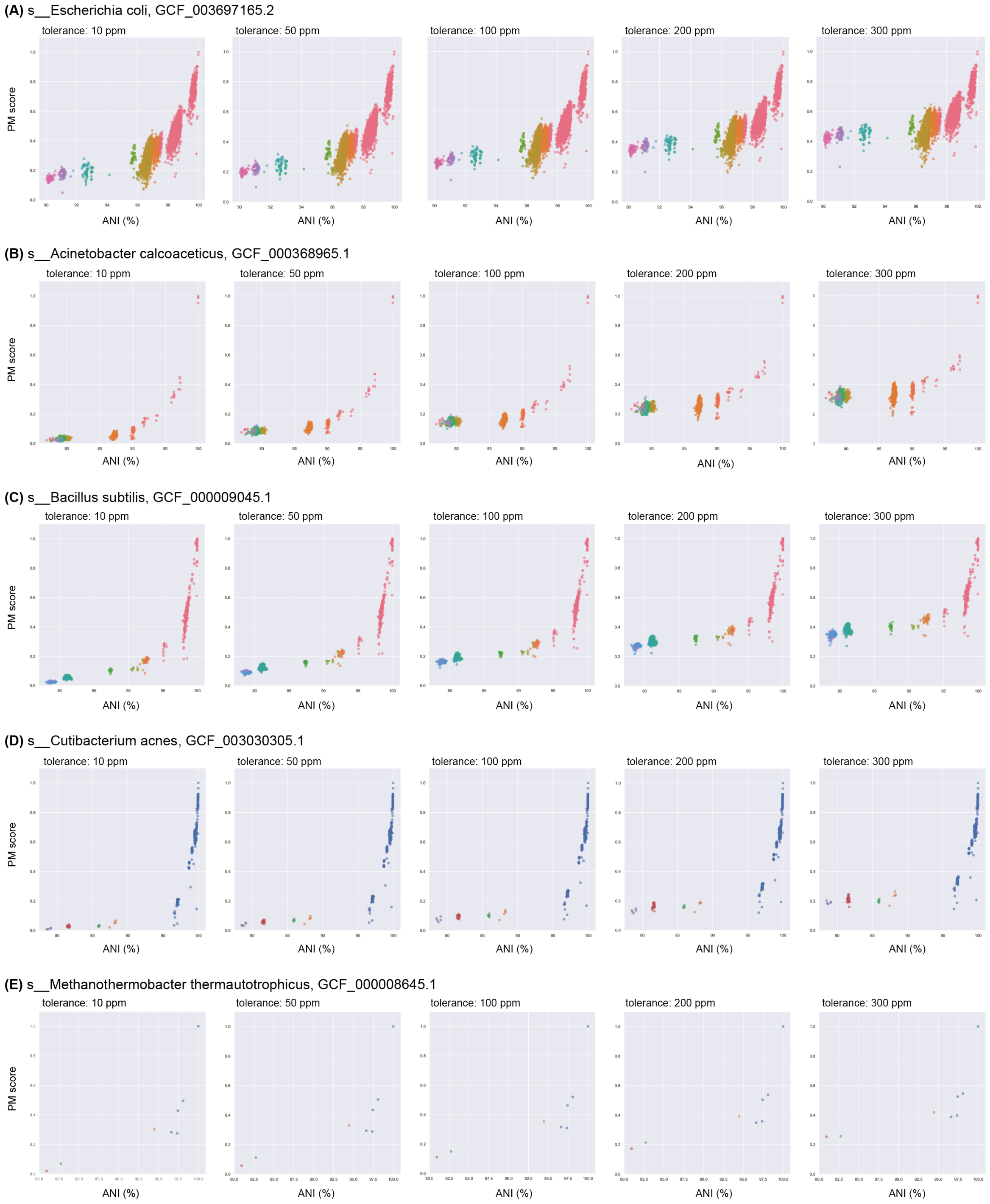

Figure S1. Relationship between average nucleotide identity (ANI) and genome-wide peak matching (PM) score. For each of the five query genomes (see panel labels for species names and RefSeq assembly accessions), PM scores with closely related genomes (references) in the GPMsDB were calculated at varying mass error tolerances for peak matching (10 to 300 ppm as shown in the facet labels). Each dot represents a query-reference genome pair, with colours indicating the taxonomy of the reference at the species level in the GTDB r95.

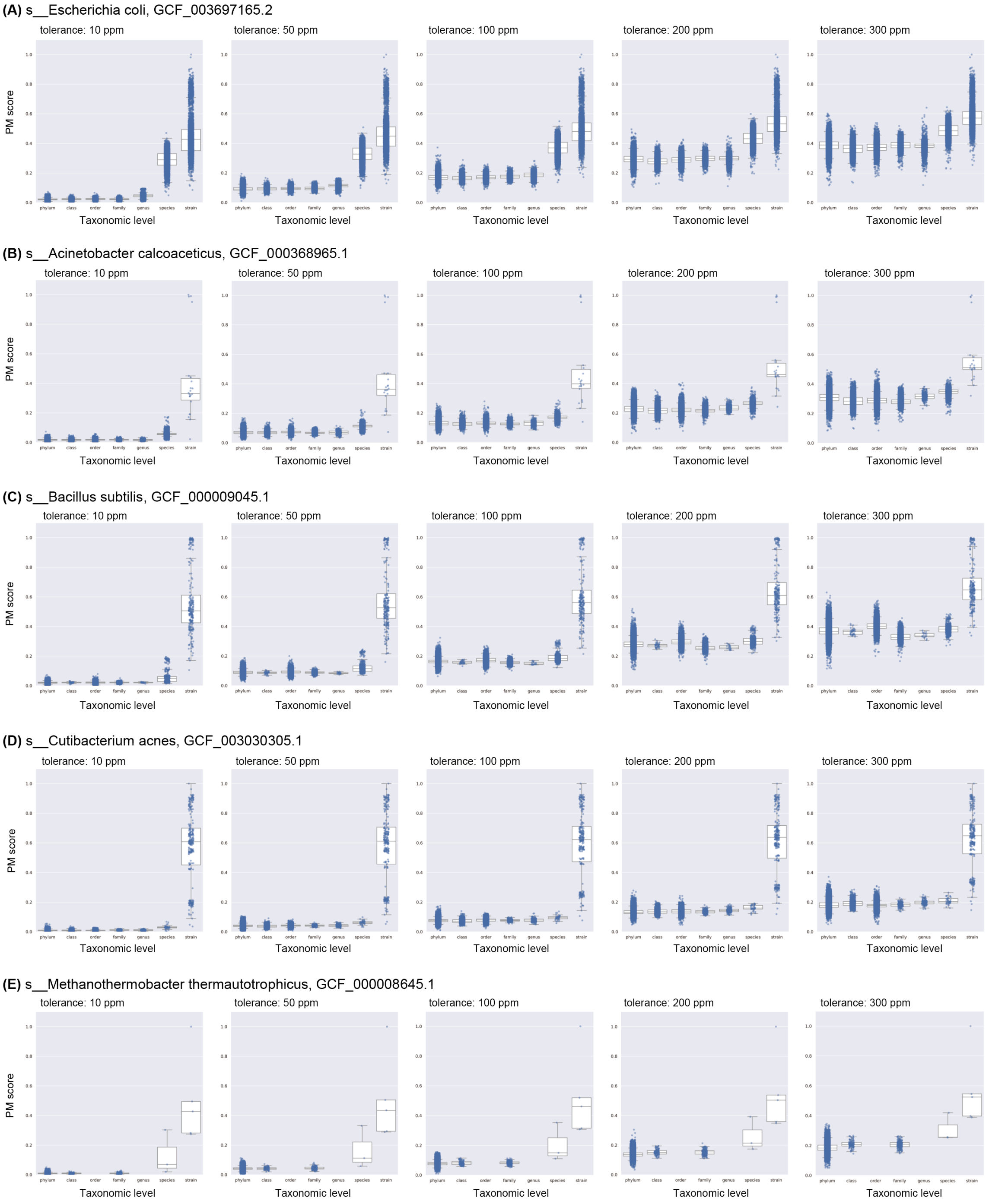

Figure S2. Relationship between taxonomic distance and genome-wide peak matching (PM) score. For each of the five query genomes (see panel labels for species names and RefSeq assembly accessions), PM scores with all genomes (references, n = 193,197) in the GPMsDB were calculated at varying mass error tolerances for peak matching (10 to 300 ppm, as shown in the facet labels) and plotted as a function of the taxonomic distance between the query and reference genomes (for example, the taxonomic level "strain" on the *x*-axis shows PM scores between the query and all references within the same species). Pairs of query and reference genomes are shown as dots, with the distribution of the quantitative data as box plots, where

the quartiles of the dataset are shown as boxes while the whiskers extend to show the rest of the distribution, except for points that were determined to be “outliers” using a method that is a function of the interquartile range.

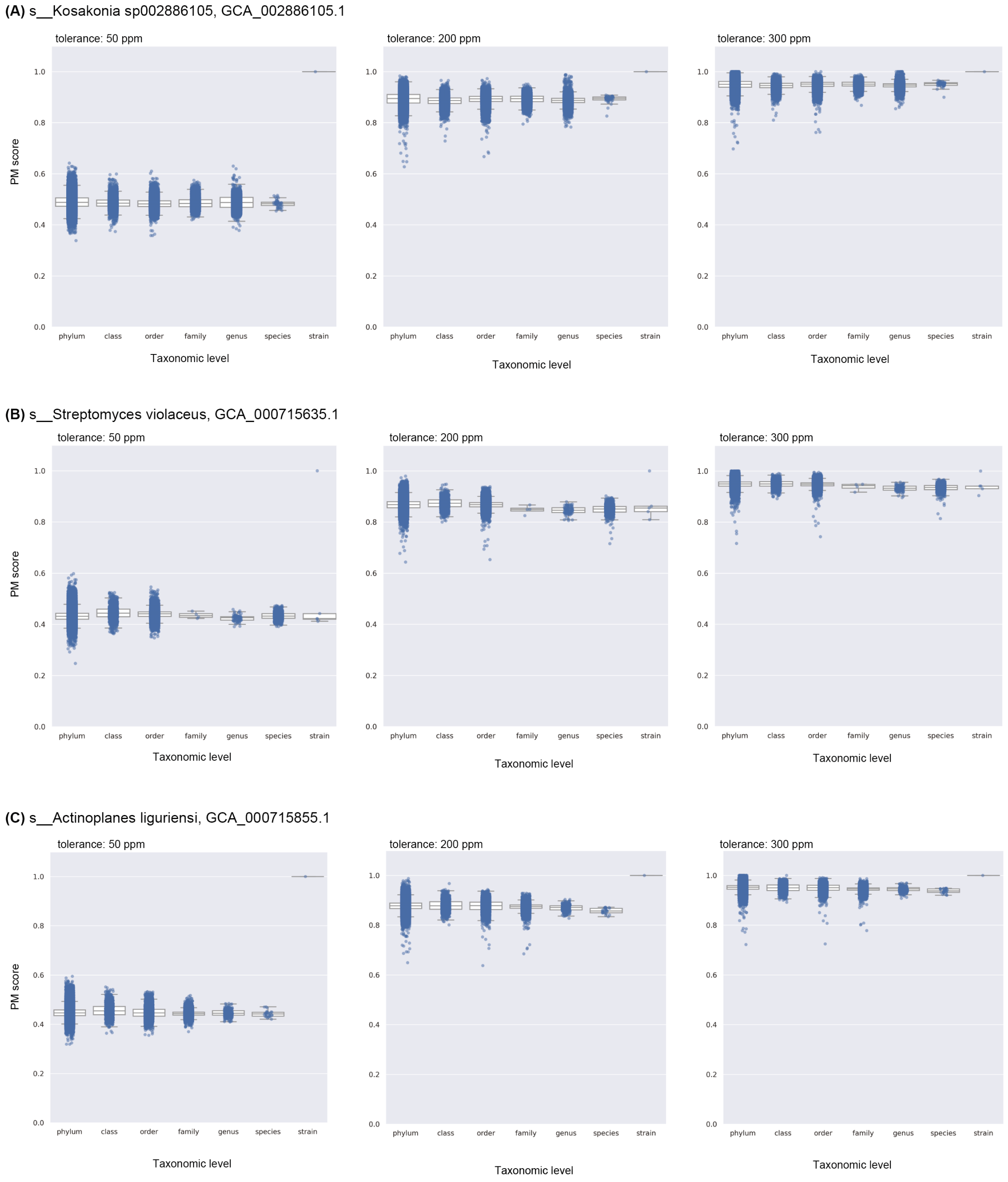

Figure S3. Relationship between taxonomic distance and genome-wide peak matching (PM) score for three genome assemblies with among the highest number of genes, or predicted mass peaks, in the GPMsDB (*Kosakonia* sp002886105: 8,428 predicted mass peaks with *m/z* values of 2,000–20,000; *Streptomyces violaceus*: 8,375*; Actinoplanes liguriensi*: 8,171). For each of the query genomes (see panel labels for species names and RefSeq assembly accessions), PM scores with all genomes (references, *n* = 193,197) in the GPMsDB were calculated at varying mass error tolerances for peak matching (10, 200 and 300 ppm, as shown in the facet labels) and plotted as a function of the taxonomic distance between the query and reference genomes (for example, the taxonomic level "strain" on the *x*-axis shows PM scores between the query

and all references within the same species). Pairs of query and reference genomes are shown as dots, with the distribution of the quantitative data as box plots, where the quartiles of the dataset are shown as boxes while the whiskers extend to show the rest of the distribution, except for points that are determined to be “outliers” using a method that is a function of the interquartile range.

(A) s__Escherichia coli, GCF_003697165.2, 1,041 genes, scoring scheme I

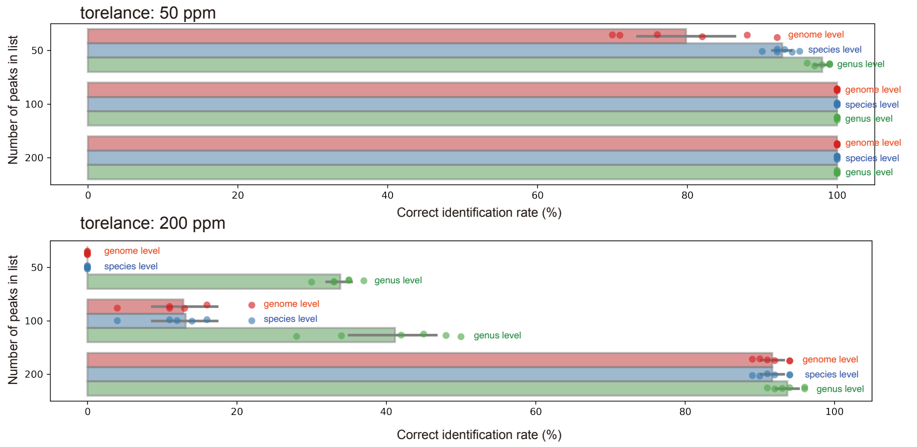

(B) s__Escherichia coli, GCF_003697165.2, 1,041 genes, scoring scheme II

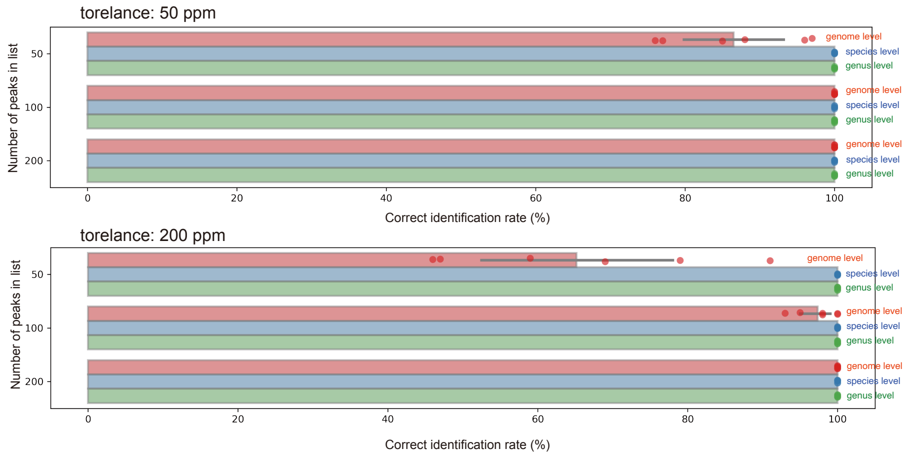

(C) s__Cutibacterium acnes, GCF_003030305.1, 453 genes, scoring schemes I and II with a tolerance of 200 ppm

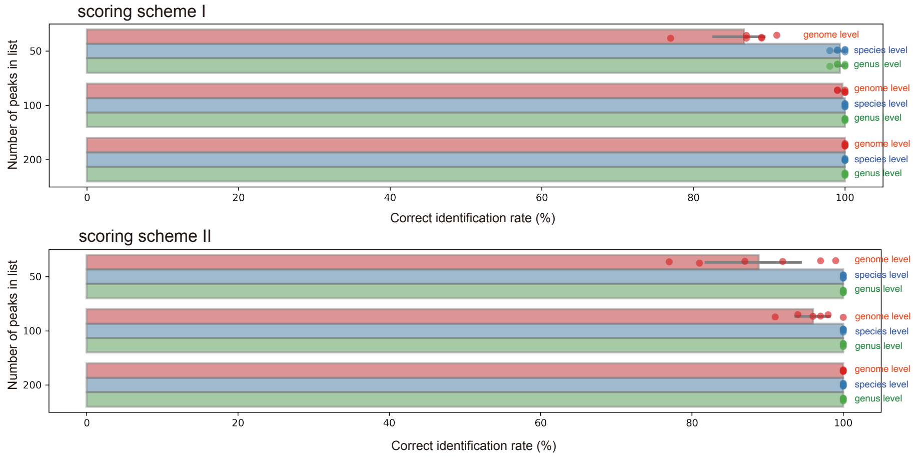

(D) s__Mycoplasmopsis columbina, GCF_900660685.1, 101 genes, scoring schemes I and II with a tolerance of 200 ppm

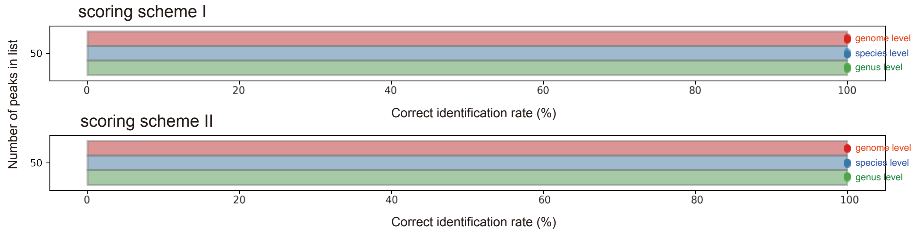

Figure S4. Effect of scoring scheme on identification accuracy as evaluated with simulated mass peak lists. (A) Identification accuracy for scoring scheme I at low (10 ppm; see plot label) and high (200 ppm) mass error tolerance for peak lists with varying numbers of total peaks (50, 100 and 200 as indicated on the *y*-axis), as evaluated with *E. coli* (RefSeq assembly accession GCF_003697165.2). For this analysis, five sets of peak lists (100 lists per set) of theoretically expected mass peaks were generated for a given number of total peaks. For each peak list, the best-matching reference in the database was identified based on PM scoring scheme I. Identification accuracy (that is, the number of correctly identified peak lists, out of 100) was then calculated at different taxonomic levels (genome level: upper-bar with red, species level: middle-bar with blue, genus level: bottom-bar with green) for each set of peak lists. The data are shown as the mean (bars) and standard deviation (error bars) across the five sets for a given combination of total peaks (*y*-axis) and taxonomic level (fill colour); symbols show identification accuracy for each of the five sets. (B) Same as for panel A, using scoring scheme II for calculating PM scores. (C and D) Comparison of scoring schemes I and II for two genomes with a low number of genes, as indicated in the panel labels. For this analysis, peak matching was performed at a mass error tolerance of 200 ppm. For panels A and B, the data are shown as the mean and standard deviation of the identification accuracy for five sets of peak lists.

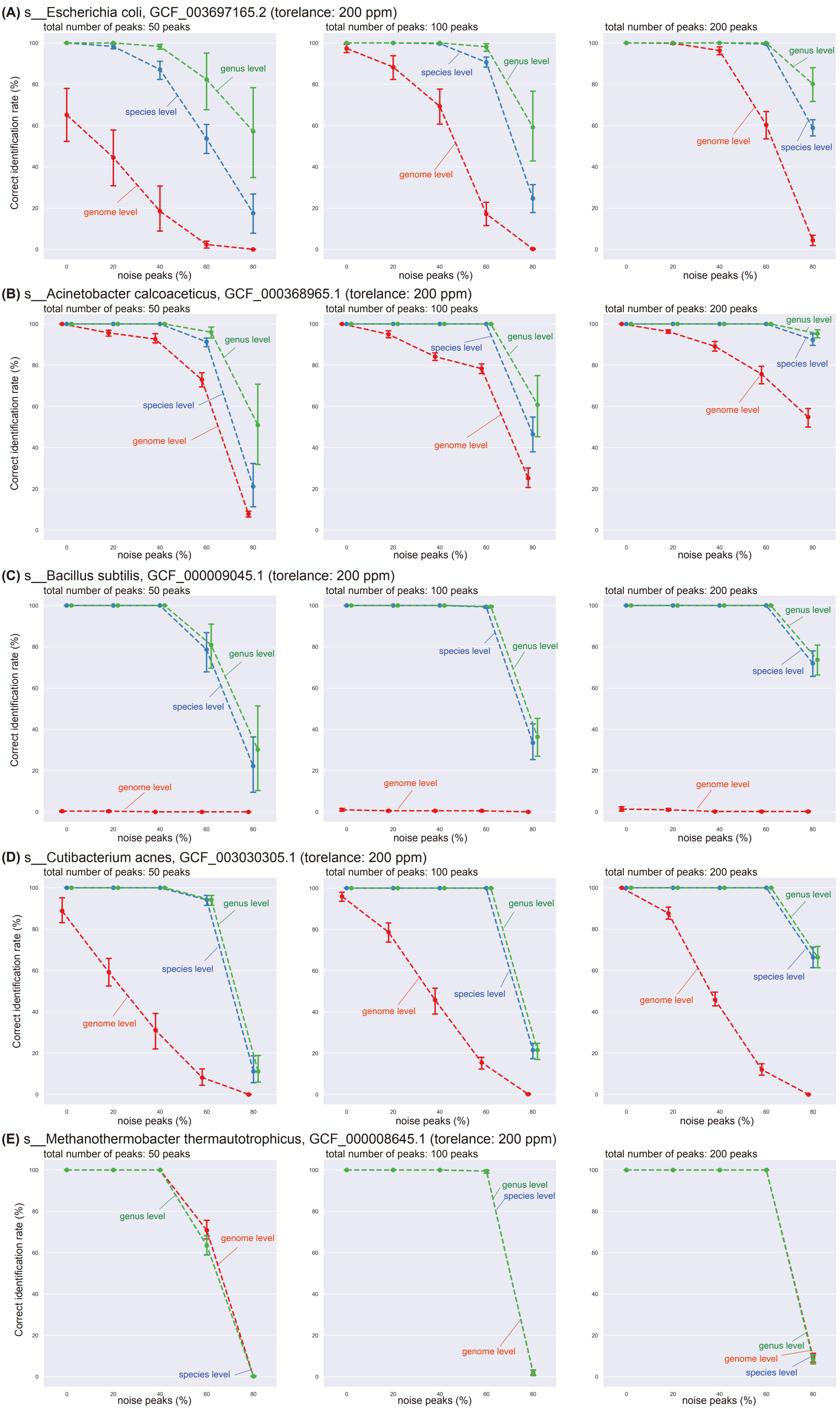
(A) s__Escherichia coli, GCF_003697165.2 (torelance: 200 ppm)
total number of peaks: 50 peaks
total number of peaks: 100 peaks
total number of peaks: 200 peaks
Correct identification rate (%)
noise peaks (%)
genus level
species level
genome level
(B) s__Acinetobacter calcoaceticus, GCF_000368965.1 (torelance: 200 ppm)
total number of peaks: 50 peaks
total number of peaks: 100 peaks
total number of peaks: 200 peaks
Correct identification rate (%)
noise peaks (%)
genus level
species level
genome level
(C) s__Bacillus subtilis, GCF_000009045.1 (torelance: 200 ppm)
total number of peaks: 50 peaks
total number of peaks: 100 peaks
total number of peaks: 200 peaks
Correct identification rate (%)
noise peaks (%)
genus level
species level
genome level
(D) s__Cutibacterium acnes, GCF_003030305.1 (torelance: 200 ppm)
total number of peaks: 50 peaks
total number of peaks: 100 peaks
total number of peaks: 200 peaks
Correct identification rate (%)
noise peaks (%)
genus level
species level
genome level
(E) s__Methanothermobacter thermautotrophicus, GCF_000008645.1 (torelance: 200 ppm)
total number of peaks: 50 peaks
total number of peaks: 100 peaks
total number of peaks: 200 peaks
Correct identification rate (%)
noise peaks (%)
genus level
species level
genome level

Figure S5. Accuracy of identification using scoring scheme I for simulated peak lists with varying degrees of "noise". For this analysis, simulated peak lists consisted of theoretically expected mass peaks and peaks with random *m/z* values, mimicking noisy measurements; random peaks accounted for 0 to 80% of total peaks in a given list (*x*-axis), and the total number of peaks per list varied between 50, 100 and 200 (see facet labels). For each combination of noise level and total number of peaks, five sets of peak lists were generated, with each set containing 100 peak lists. For each peak list, the best-matching reference in the GPMsDB was identified at a mass error tolerance of 200 ppm and ranking matched peak lists using scoring scheme I. Identification accuracy (that is, the number of correctly identified peak lists, out of 100) was then calculated at different taxonomic levels (genome level: red, species level: blue, genus level: green) for each set of peak lists. The data are shown as the mean (symbols) and standard deviation (error bars) across the five peak list sets for each combination of noise level (x-axis), total number of peaks (facet labels) and taxonomic level (symbol and line colours).

(A) s__Escherichia coli, GCF_003697165.2

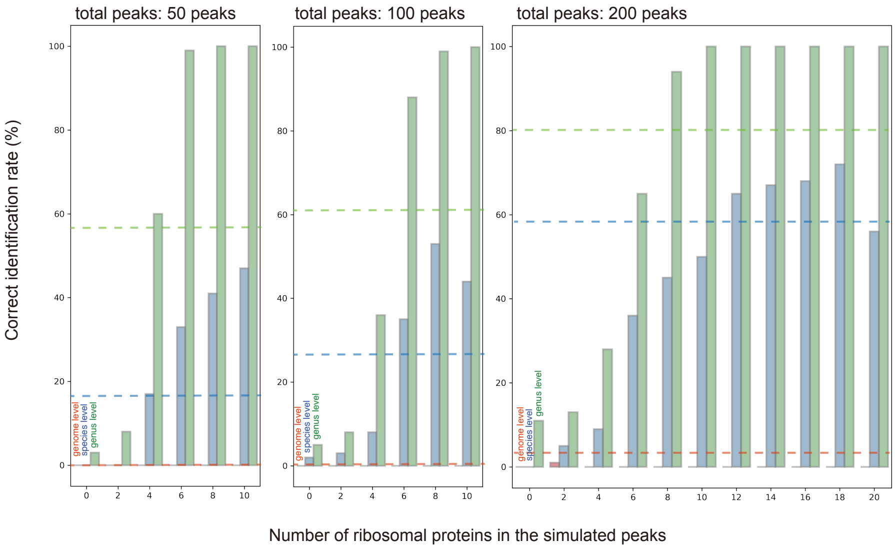

(B) s__Acinetobacter calcoaceticus, GCF_000368965.1

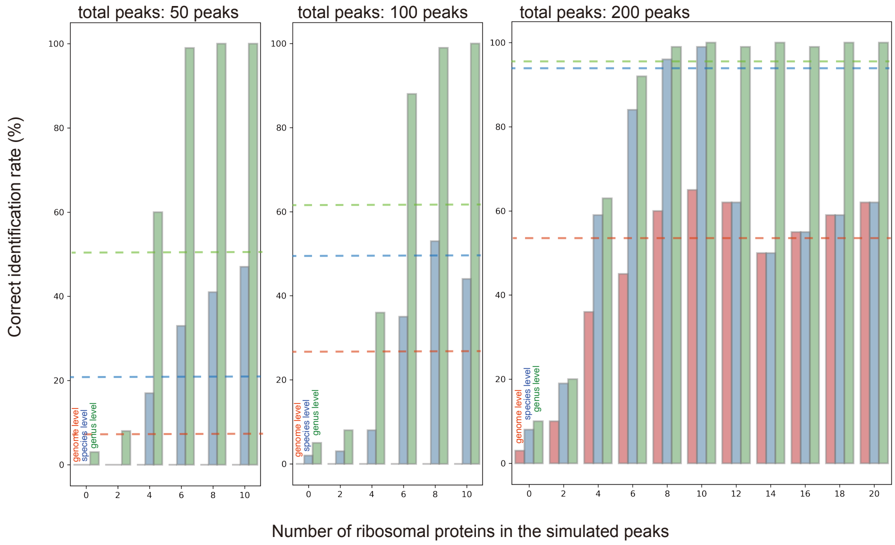

(C) s__Bacillus subtilis, GCF_000009045.1

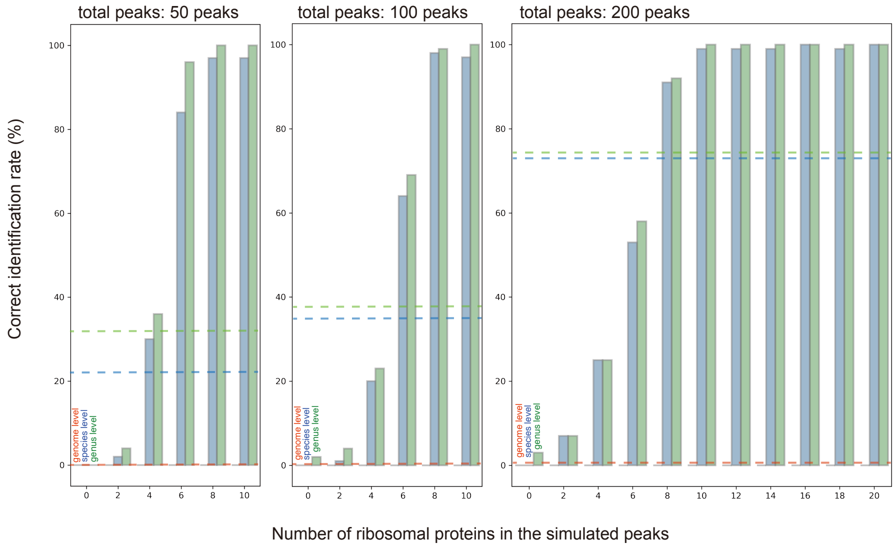

(D) s__Cutibacterium acnes, GCF_003030305.1

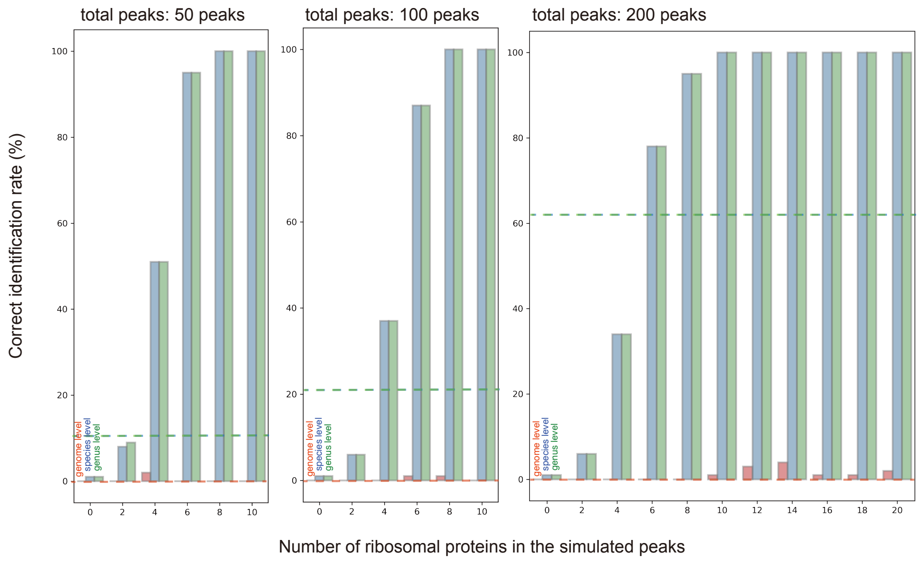

(E) s__Methanothermobacter thermautotrophicus, GCF_000008645.1

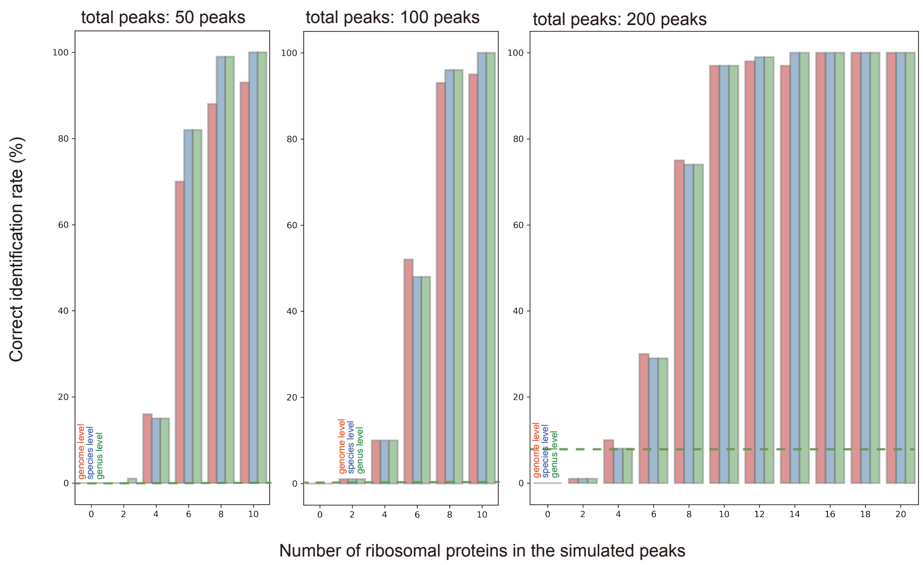

Figure S6. Accuracy of identification using scoring scheme III for simulated peak lists containing varying numbers of ribosomal protein peaks. For this analysis, simulated peak lists consisted of theoretically expected mass peaks with varying numbers of ribosomal protein mass peaks (see *x*-axis); mass peaks with random *m*/*z* values accounted for 80% of total peaks, and the total number of peaks per list varied among 50, 100 and 200 (see facet labels). For each combination of number of ribosomal protein peaks and total number of peaks, five sets of peak lists were generated, with each set containing 100 peak lists. For each peak list, the best-matching reference in the GPMsDB was identified at a mass error tolerance of 200 ppm, and matched peak lists were ranked using scoring scheme II. Identification accuracy (that is, the number of correctly identified peak lists out of 100) was then calculated at different taxonomic levels (genome level: left-bar with red, species level: middle-bar with blue, genus level: right-bar with green) for each set of peak lists. The data are shown as the mean (symbols) and standard

deviation (error bars) across the five peak list sets for each combination of number of ribosomal protein peaks (*x*-axis), total number of peaks (facet labels) and taxonomic level (symbol and line colours). Dashed horizontal lines show the average identification accuracy achieved using scoring scheme I. Note that the number of genes shown in the title of each panel (A-D) represents the number of genes encoding proteins in the range of 2,000–15,000 Da.

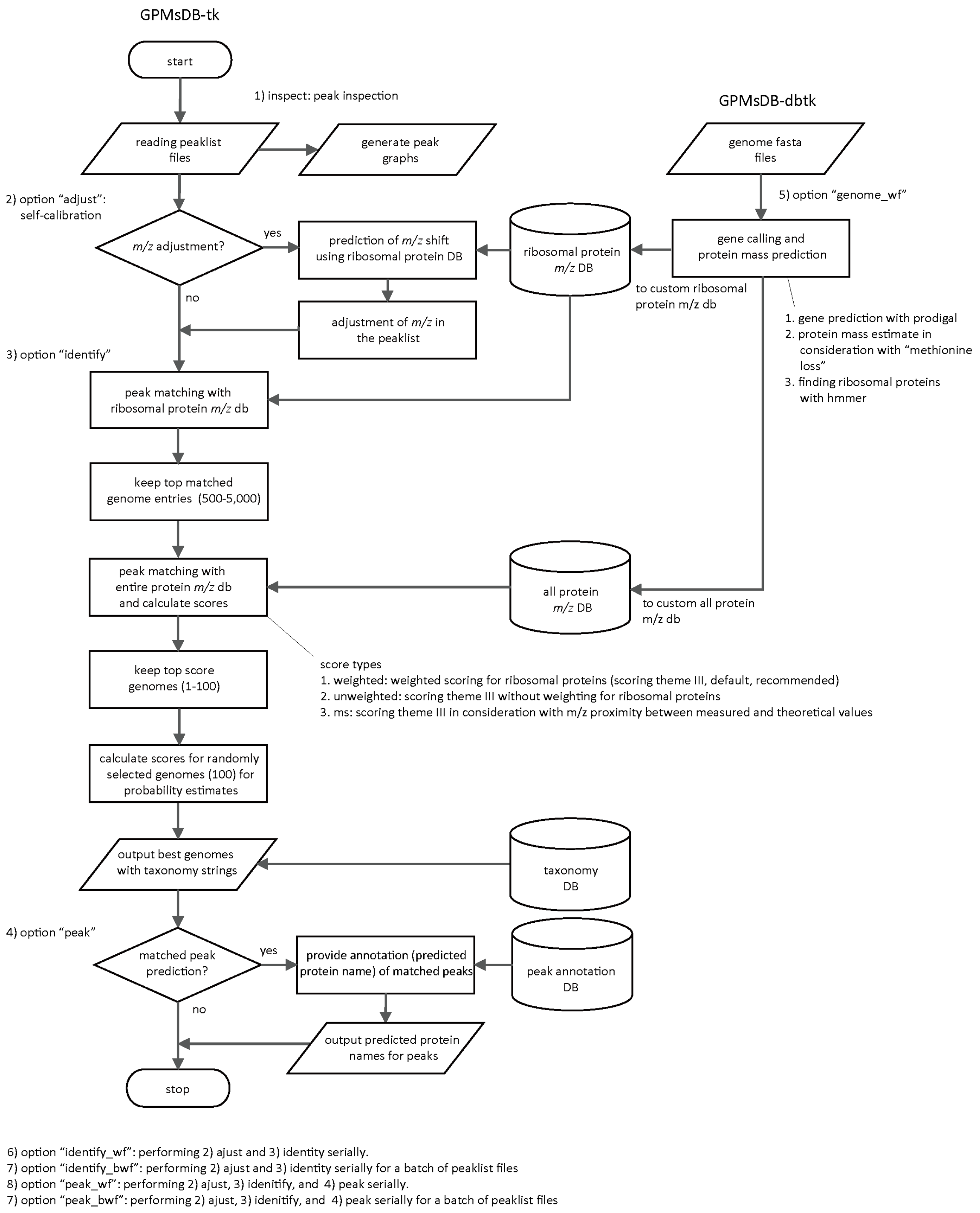

Figure S7. Schematic of the GPMsDB-tk.

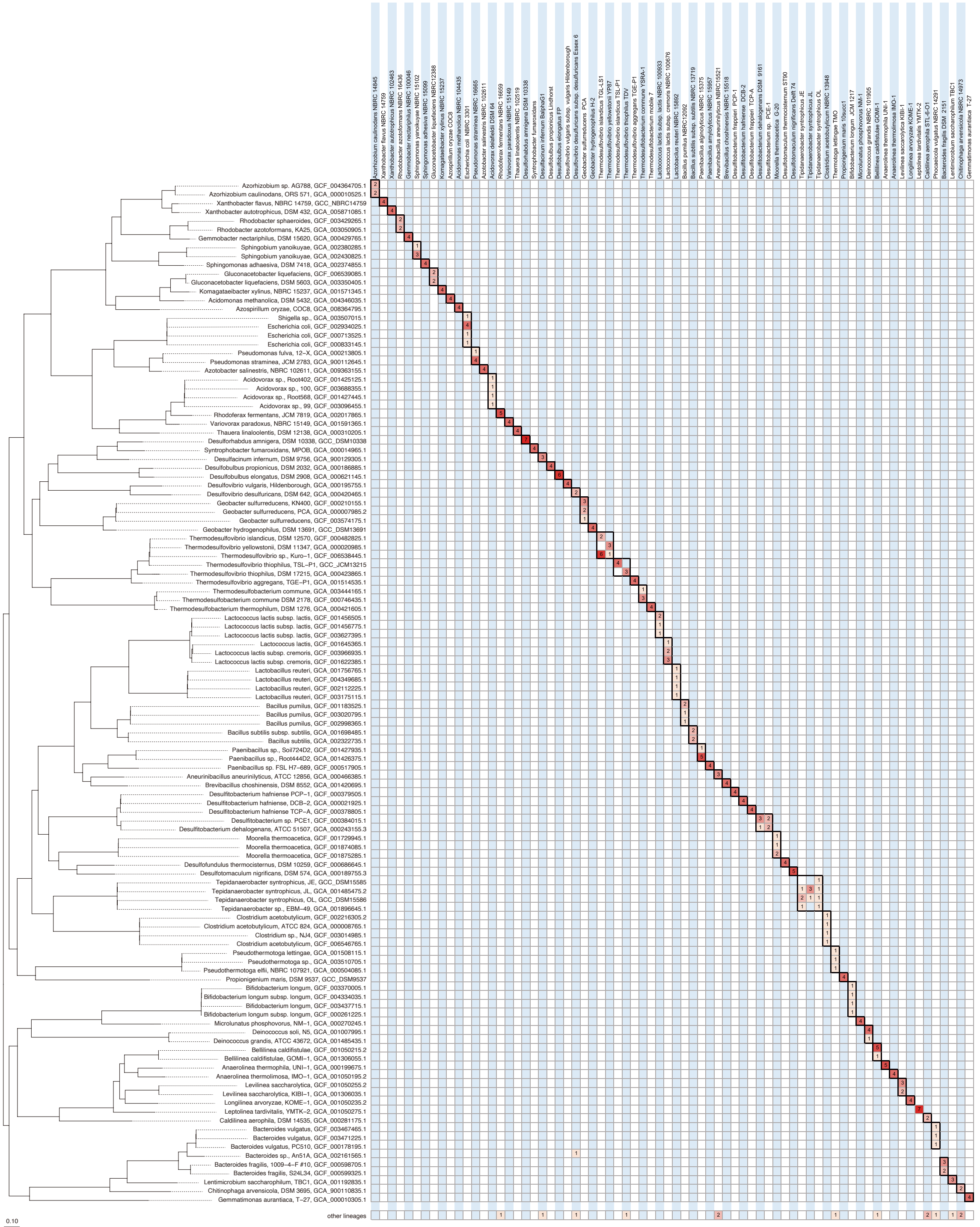

Figure S8. Identification results for MALDI-TOF MS peak lists of 71 bacterial reference strains. For each of the strains, at least four MALDI-TOF MS determinations were performed, and the best-matching hits were identified using GPMsDB-tk, with scoring scheme III and a mass error tolerance of 200 ppm. All genomes in the GPMsDB-tk were searched by specifying command line option -a as "all". Values in the heatmap indicate the number of repeated determinations assigned to the corresponding genome in the phylogenetic tree. Tiles with thick black borders indicate correct identification at least at the species level. Best-matching hits other than the genomes included in the tree are grouped as "other lineages" at the bottom of the heatmap.

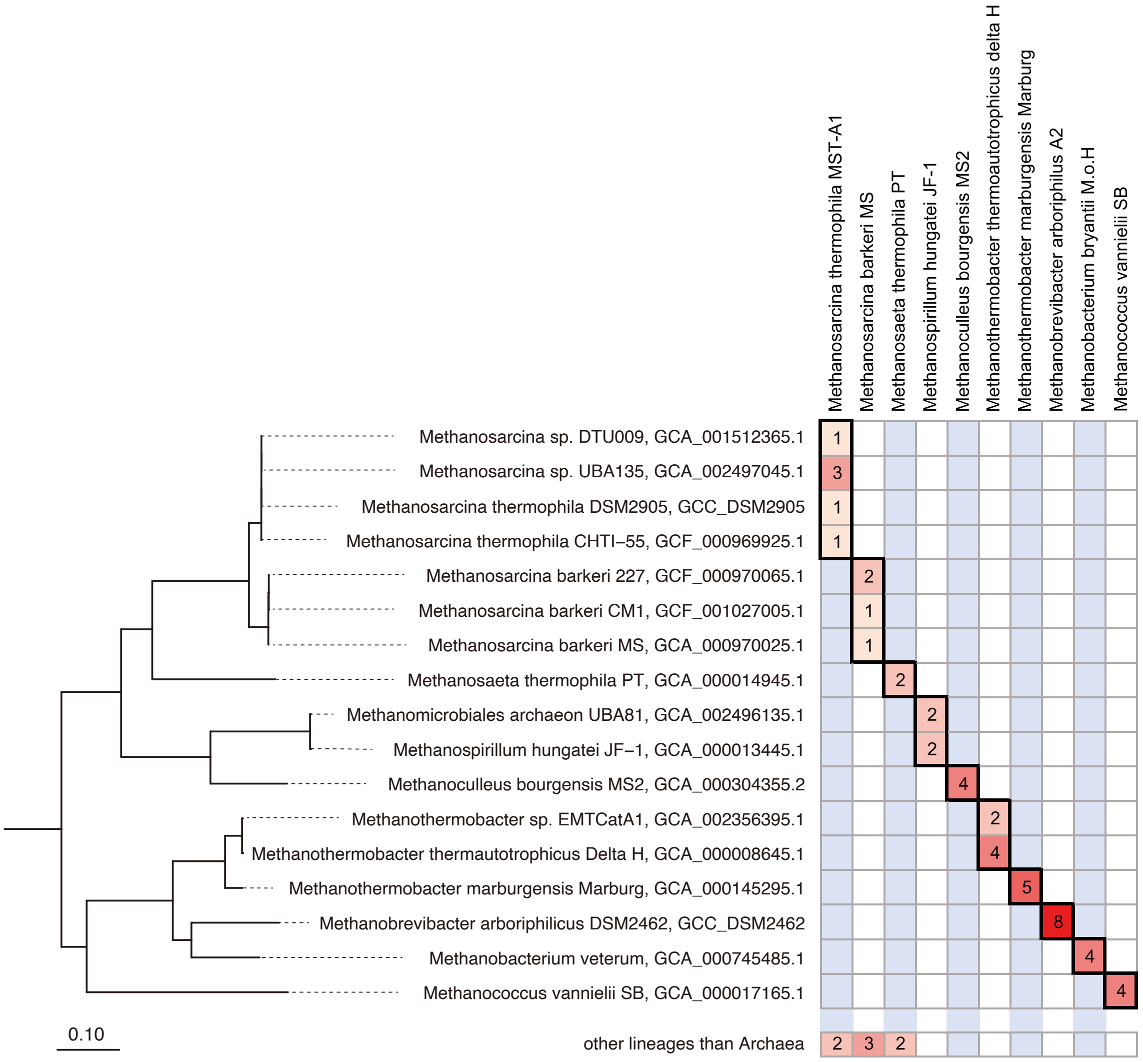

Figure S9. Identification results for MALDI-TOF MS peak lists of 10 archaeal strains. For each of the strains, at least four MALDI-TOF MS determinations were performed, and the best-matching hits were identified using GPMsDB-tk, with scoring scheme III and a mass error tolerance of 200 ppm. All genomes in the GPMsDB-tk were searched by specifying command line option -a as "all". Values in the heatmap indicate the number of repeated determinations assigned to the corresponding genome in the phylogenetic tree. Tiles with thick black borders indicate correct identification at least at the species level. Best-matching hits other than the genomes included in the tree are grouped as "other lineages" at the bottom of the heatmap.

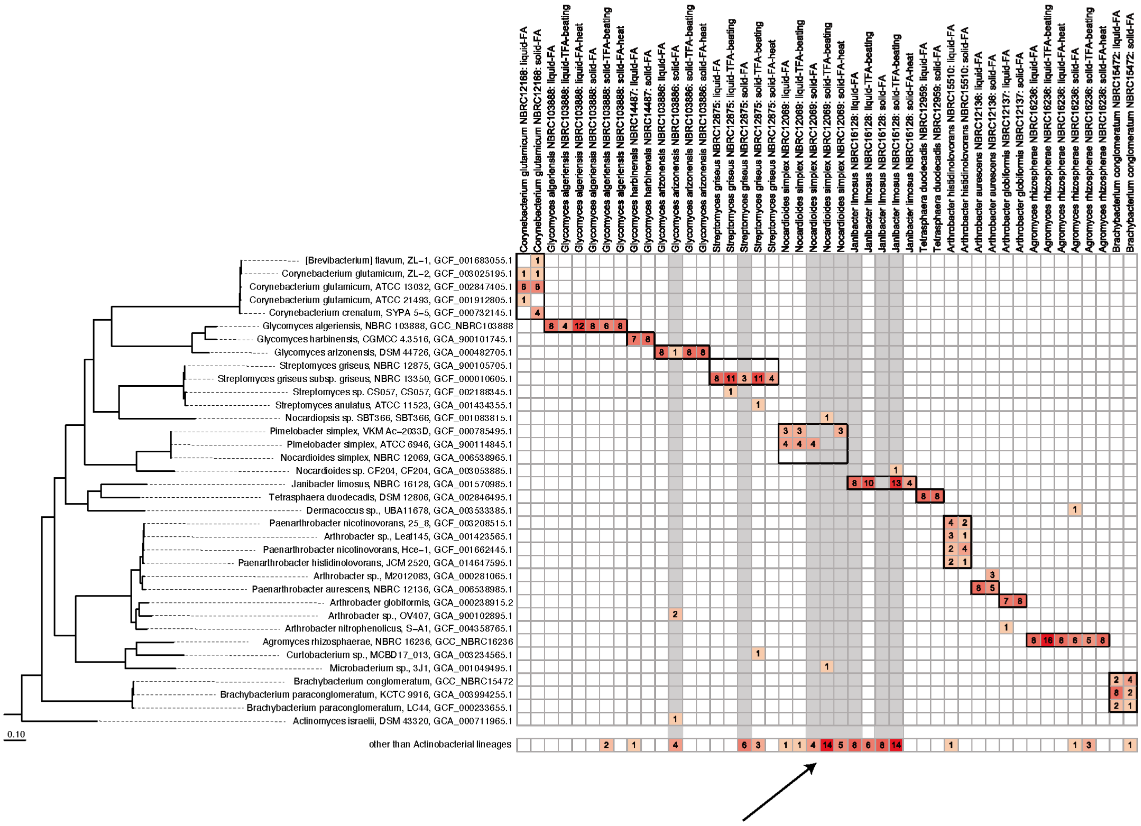

Table summarizing the result of MALDI-TOF MS identification for the four actinobacterial strains which seem resistant to cell lysis.

Organism	Culture type	Medium	FA method: number of correct identification/measurement replicates	FA method: correct identification (%)	TFA-beating method: number of correct identification/measurement replicates	TFA-beating method: correct identification (%)	FA-heating method: number of correct identification/measurement replicates	FA-heating method: correct identification (%)
Glycomyces arizonensis NBRC103886	liquid-medium	227	4/4	100%	4/4	100%	4/4	100%
		802	4/4	100%	4/4	100%	4/4	100%
	solid-medium	227	4/4	50%	4/4	100%	4/4	100%
		802	0/4	0%	4/4	100%	4/4	100%
Streptomyces griseus NBRC12875	liquid-medium	227	4/4	100%	6/6	100%	not tested	not tested
		802	4/4	100%	6/6	100%	not tested	not tested
	solid-medium	227	3/4	75%	12/12	100%	not tested	not tested
		802	1/5	20%	0/5	0%	4/4	100%
Nocardioides simplex NBRC12069	liquid-medium	230	4/4	100%	4/4	100%	not tested	not tested
		802	3/4	75%	3/4	75%	not tested	not tested
	solid-medium	230	0/4	0%	0/8	0%	0/4	0%
		802	4/4	100%	0/8	0%	3/4	75%
Janibacter limosus NBRC16128	liquid-medium	230	0/8	0%	6/8	75%	not tested	not tested
		802	8/8	100%	8/8	100%	not tested	not tested
	solid-medium	230	0/4	0%	11/20	55%	not tested	not tested
		802	0/4	0%	5/8	63%	4/4	100%

Figure S10. Identification results for MALDI-TOF MS peak lists of 13 strains of *Actinomycetota*. Culture conditions and sample pretreatment methods are indicated in the column headers. For each of the strains, at least four MALDI-TOF MS determinations were performed, and the best-matching hits were identified using GPMsDB-tk, with scoring scheme III and a mass error tolerance of 200 ppm. All genomes in the GPMsDB-tk were searched by specifying command line option -a as “all”. Values in the heatmap indicate the number of repeated determinations assigned to the corresponding genome in the phylogenetic tree. Tiles with thick black borders indicate correct identification at least at the species level. Best-matching hits other than the genomes included in the tree are grouped as “other lineages” at the bottom of the heatmap. The table at the bottom shows the correctness of the MALDI-TOF MS-based identification for *Glycomyces arizonensis*, *Streptomyces griseus*, *Nocardioides simplex*, and *Janibacter limosus* cells under varying cultivation and pretreatment conditions.

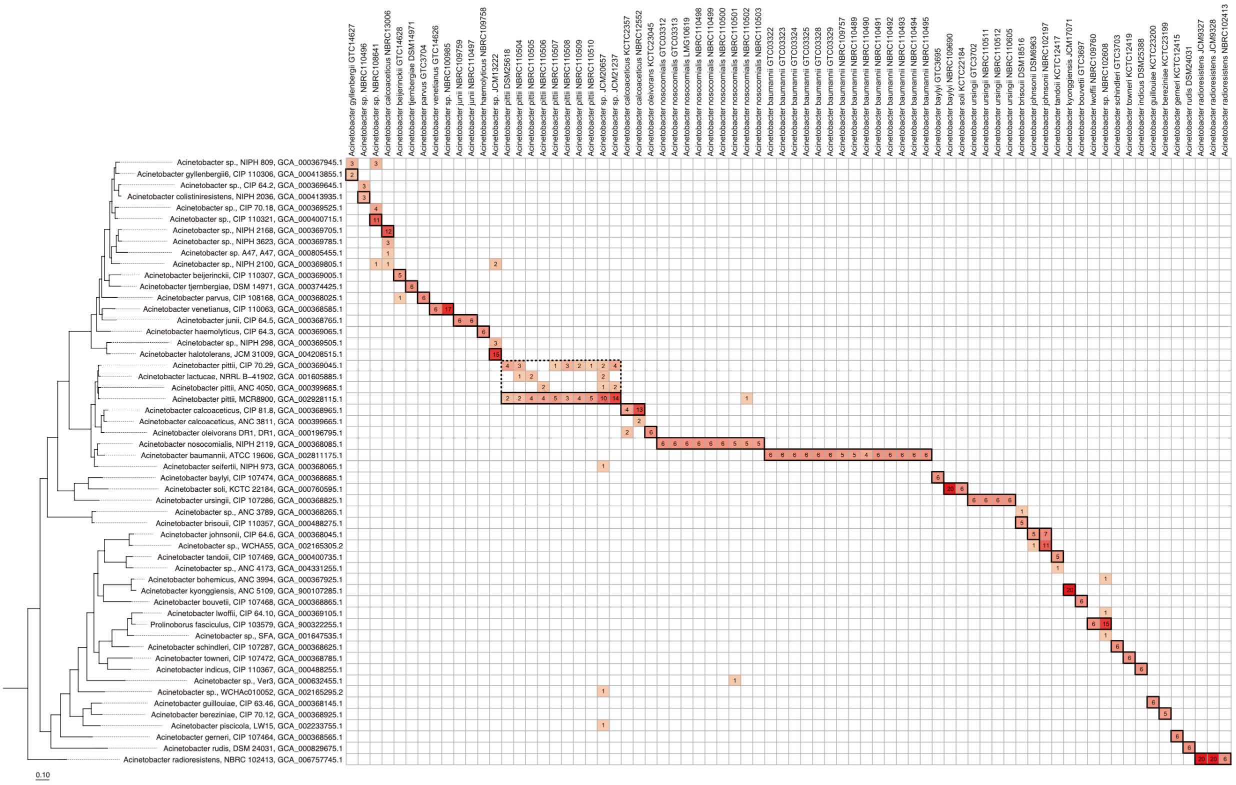

Figure S11. Identification results for MALDI-TOF MS peak lists for 74 different *Acinetobacter* strains. Peak lists (n = 616) were obtained from NBRC, NITE, Japan (SAC001). The data were analysed and visualized as shown in Figure S8, except that only representative genomes in the GPMsDB were searched by specifying command line option -a as “reps”.

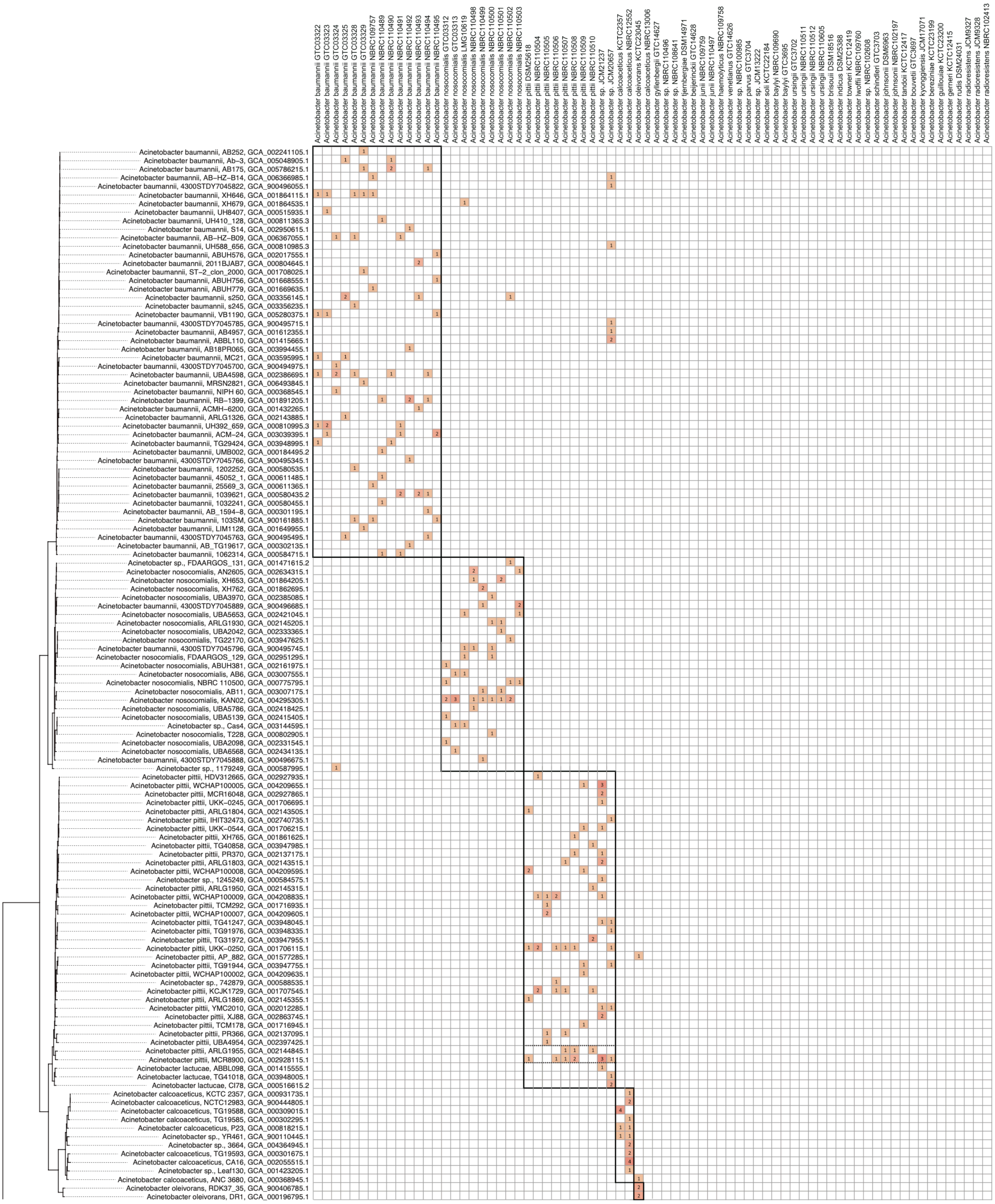

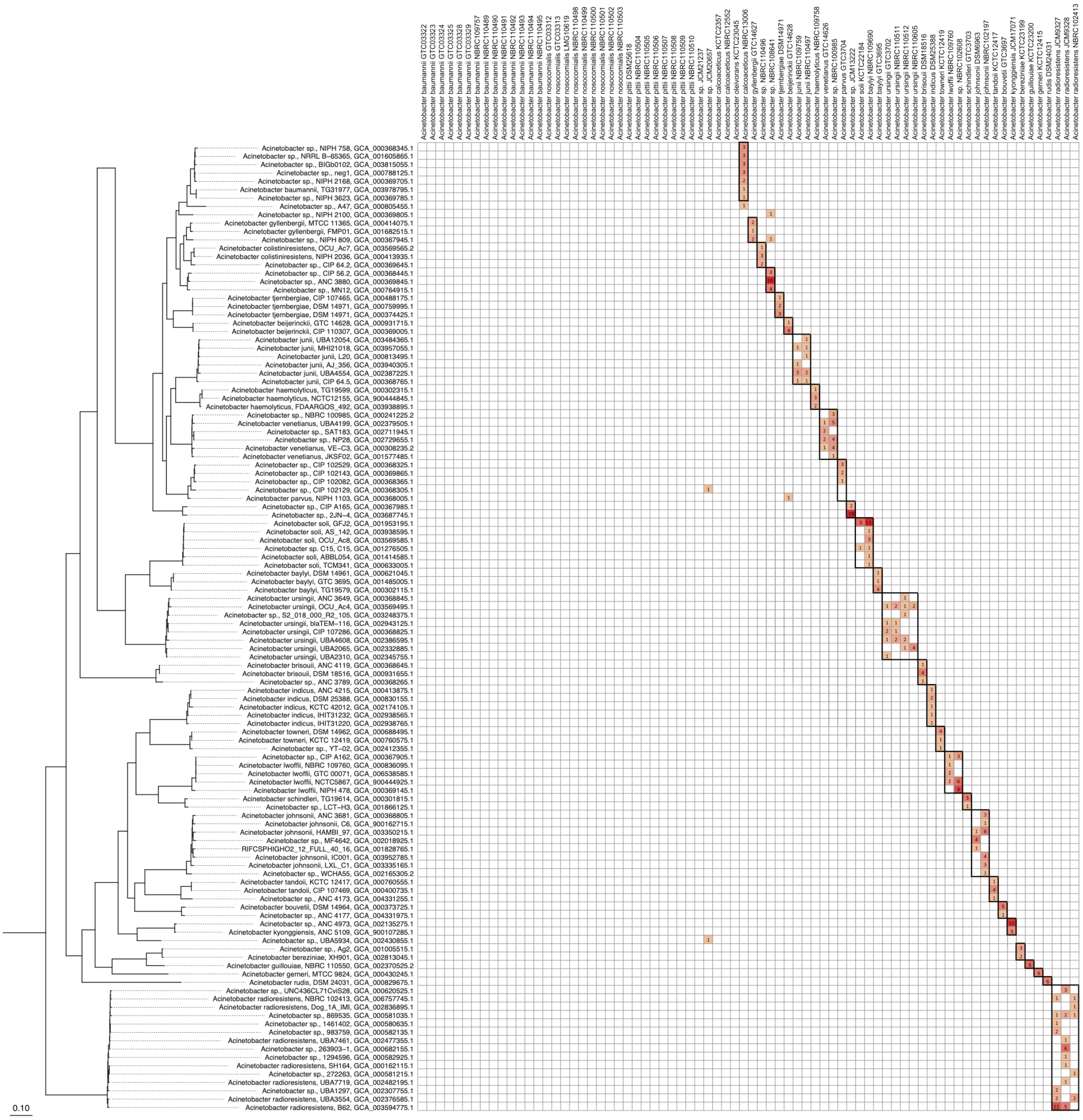

Figure S12. Same as for Figure S11, except that all genomes in the GPMsDB were searched by specifying command line option -a as “all”

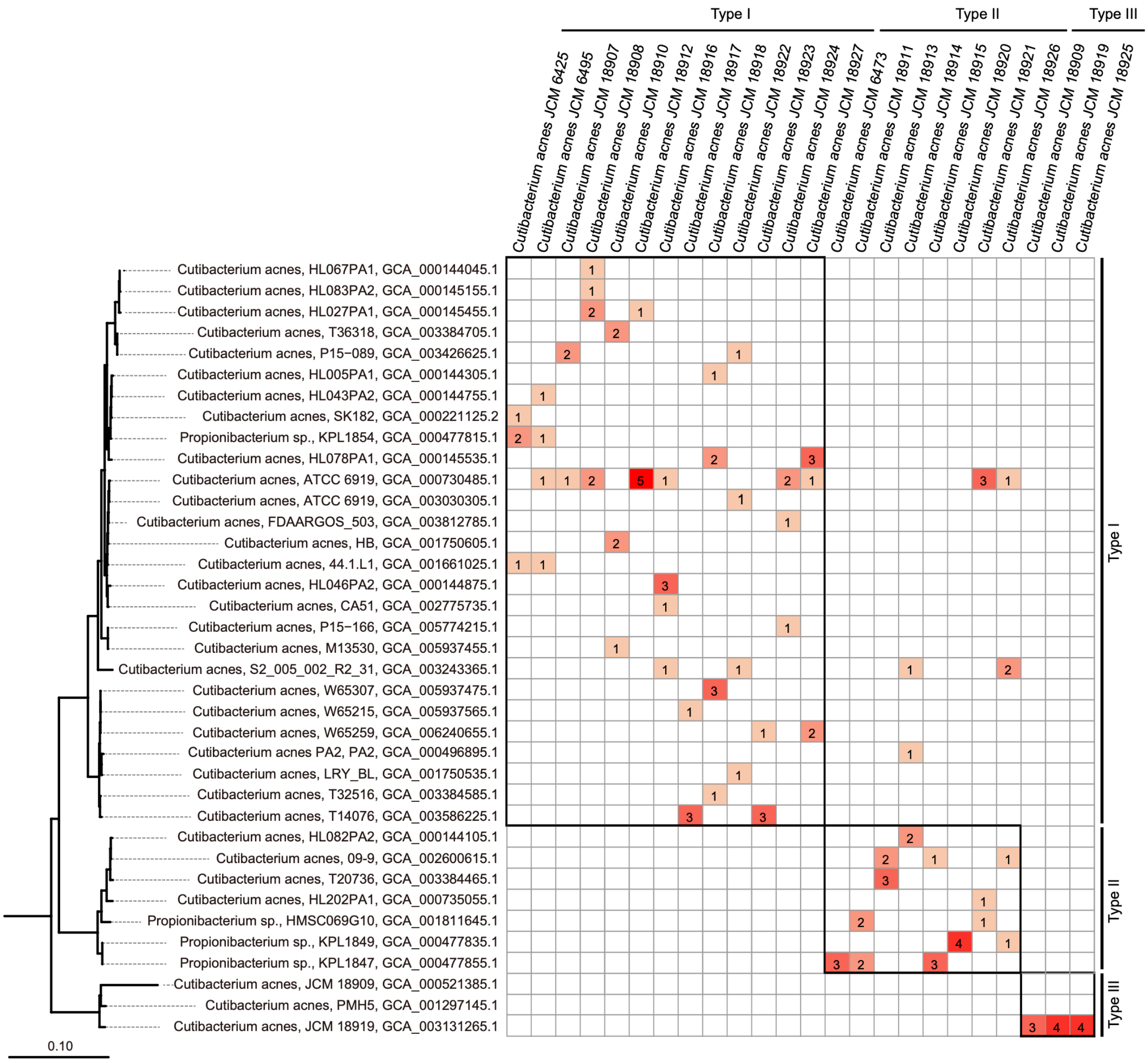

Figure S13. Identification results for MALDI-TOF MS peak lists for 24 different strains of *Cutibacterium acnes*. Peak lists (n=118) were obtained from Teramoto et al. (2019). The data were analysed and visualized as shown in Figure S8.
